# Optoelectronic Synapse Behaviors in Tb^3+^ and Al^3+^ Co‐Doped CaSnO_3_ with Long‐Persistent Luminescence

**DOI:** 10.1002/advs.202402848

**Published:** 2024-06-26

**Authors:** Sangwon Wi, Minjae Jeong, Kwanchul Lee, Yunsang Lee

**Affiliations:** ^1^ Department of Physics and Integrative Institute of Basic Sciences Soongsil University Seoul 06978 Republic of Korea

**Keywords:** artificial synapse, long persistent luminescence, synaptic behavior

## Abstract

Neuromorphic computation draws inspiration from the remarkable features of the human brain including low energy consumption, parallelism, adaptivity, cognitive functions, and learning ability. These qualities hold the promise of unlocking groundbreaking computational techniques that surpass the limitations of traditional computing systems. This paper reports a remarkable photo‐synaptic behavior in the field of rare earth ion‐doped luminescent oxides by using long‐persistent luminescence (LPL). This system utilizes electron trap states to regulate the synaptic behavior, operating through a fundamentally different mechanism from that of electronic‐based synaptic devices. To realize this strategy, Tb^3+^ doped CaSnO_3_, which shows a significant LPL property under UV‐light excitation, is prepared. The luminescent system shows key neuromorphic characteristics such as paired‐pulse facilitation, pulse‐number/timing dependent potentiation, and pulse‐number/timing dependent short‐ to long‐term plasticity transition, which are required for realizing synaptic devices. This feature expands the way for advanced neuromorphic technologies employing light stimuli.

## Graphical abstract

The principal synaptic features in biological synapses are successfully replicated in Tb^3+^ and Al^3+^ co‐doped CaSnO_3_ by utilizing long‐persistent luminescence. Upon the introduction of square‐wave UV pulses, this sample exhibits a sequential increase in photoluminescence intensity with both pulse number and frequency. These findings hold the potential for the development of photonic artificial synapses using luminescent oxides.

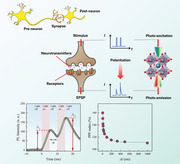

## 1 Introduction

Neuromorphic computing holds the promise of overcoming the limitations posed by the von Neumann bottleneck that plagues current mainstream computing architectures.^[^ ^1^ ^]^ This is primarily due to its ability to self‐adaptive learning and highly parallel computation, coupled with significantly reduced energy consumption. To realize this potential, the development of synaptic devices that closely emulate the biological synapsis is of paramount importance, and this new type of devices are the foundational components for neuromorphic computing systems.^[^ ^1^ ^]^ Recent strides in optoelectronic devices have provided valuable inspiration, leading to the integration of light‐based mechanisms into synaptic devices, which offer a host of benefits including broad bandwidth, minimal resistance‐capacitance delays, negligible power losses, and the capacity for global regulation across multiple synaptic elements (i.e., homeoplasticity).^[^ ^1^ ^]^

On the other hand, luminescent materials with rare earth (RE) ion doping have been explored in various fields of lightening and optical‐based sensing, such as temperature and pressure sensing, driven by their versatile nature.^[^ ^2^ ^]^ Among them, long‐persistent luminescence (LPL) materials exhibit luminescence from seconds to hours after turning off the excitation sources. Especially, RE ion‐based LPL materials have been developed intensively due to their exceptional performance and efficiency.^[^ ^3^ ^]^ It is worthwhile to mention that under the LPL behavior, the luminescent signal can be enhanced by serial photoexcitation pulses. Therefore, we suggest that luminescent materials utilizing LPL can be highly suitable and efficient for developing photonic‐based synaptic devices. Moreover, the incorporation of luminescent materials into light sources and sensors has already been successfully achieved in various applications such as lighting, displays, and solar cells. Consequently, LPL‐based synaptic devices hold promise for compatibility with existing devices. However, the exploration of this concept remains uncharted territory.

In this work, we demonstrate that Tb^3+^ and Al^3+^ co‐doped CaSnO_3_ (CSO:Tb/Al) perovskite oxides with a good LPL property under UV‐excitation can be used for realizing an optoelectronic synaptic device. We suggest that CaSnO_3_ is a promising candidate to realize our strategy. We considered three key aspects for selecting the appropriate LPL material. First, the material should exhibit significant LPL properties. Second, it should offer ample room for design flexibility in terms of LPL properties. Lastly, it should possess a significantly wide bandgap and chemical stability. We suggest that CaSnO_3_ should be a promising candidate to meet these criteria due to its wide‐bandgap, high thermal stability, and prominent LPL properties with RE ion doping.^[^ ^4^ ^]^ Furthermore, the additional doping of Al ions to Tb^3+^ doped CaSnO_3_ (CSO:Tb) was employed to increase the amount of defects. To investigate the synaptic plasticity of CSO:Tb/Al, we examined the light‐emitting responses while varying various factors, including pulse number and pulse timing. Major synaptic behaviors such as paired‐pulse facilitation (PPF), pulse‐number/timing dependent potentiation, and pulse‐number/timing dependent short‐ to long‐term plasticity transition were successfully identified. In this study, we focused on the potential and characteristics of the materials as LPL‐based synaptic material in this work, as an early stage of this concept. Finally, by using the luminescent synaptic behaviors of CSO:Tb/Al, we demonstrated two neural network (NN) applications for neuromorphic computing: handwritten digit/fashion image recognition and reservoir computing (RC).

## 2 Results

### 2.1 Sample Characteristics

**Figure 1:**
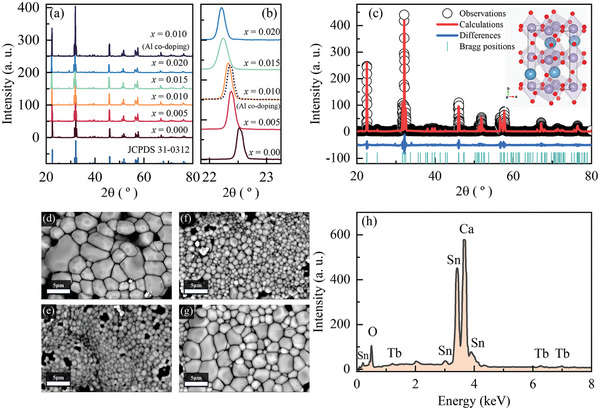
X‐ray diffraction of CSO:Tb and CSO:Tb/Al in a range of a) 20°–80° and b) 22°–23.5°, at *x* = 0.00–0.02. The dotted line represents the CSO:Tb/Al. c) Rietveld refinement profiles of CSO:Tb/Al. SEM images of CSO:Tb at *x* = d) 0.000, e) 0.010, f) 0.020, and g) CSO:Tb/Al, respectively. h) EDS spectrum of CSO:Tb at *x* = 0.020.

First, we examined the crystal structures of CSO:Tb (doping concentration of Tb^3+^, *x* = 0–0.02) and CSO:Tb/Al (*x* = 0.01, doping concentration 0.01 of Al^3+^) using X‐ray diffraction (XRD). As shown in **Figure** **1**a, the XRD patterns of CSO:Tb and CSO:Tb/Al are nearly identical to the reference pattern of CaSnO_3_, that is, orthorhombic structure (JCPDS 31–0312).^[^ ^5^ ^]^ These results indicate that the major crystal structure of CSO:Tb and CSO:Tb/Al remained unaltered, even when the Tb^3+^ and Al^3+^ doping concentration reached 2%. Interestingly, the XRD peaks shifted to a lower angle as a function of *x* (Figure 1b). It is worthwhile to note that the ionic radius of Tb^3+^ is smaller than that of Ca^2+^ in 8‐coordinate: r(Tb^3+^) = 104 pm and r(Ca^2+^) = 112 pm.^[^ ^6^ ^]^ In this sense, the A‐site substitution of the larger Ca^2+^ with the smaller Tb^3+^ should lead to a decrease in lattice constants, but this is the opposite to our observation. We note that the substitution of trivalent Tb ions for the divalent Ca ions raises the charge imbalance. To compensate the charge imbalance, cation vacancies are created, and as a result, the lattices expand due to the repulsive forces between cations.^[^ ^7^ ^]^ Meanwhile, the XRD pattern of CSO:Tb/Al showed a higher angle shift compared with that of CSO:Tb (Figure 1b). The lattice contraction by the co‐doping of Al^3+^ can be understood in term of the smaller ionic radius of Al^3+^ than that of Ca^2+^ in 6‐coordinate: r(Al^3+^) = 53.5 pm Å and r(Ca^2+^) = 100 pm in 6‐coordinate.^[^ ^6^ ^]^

To obtain the details of the structural properties of our samples, we performed a Rietveld refinement analysis of the acquired XRD patterns (Figure 1c).^[^ ^8^ ^]^ The *2θ* range of the XRD patterns used for the Rietveld refinement was 20°–80°, where all major Bragg peaks of CaSnO_3_ were included. We specified the crystal structure as orthorhombic *Pnma*. The weighted profile R‐factors (R_wp_) were calculated in the range of 7.30–18.75, and the goodness of fit (χ^2^) values were <2.0, indicating that our refinement results are reasonable.^[^ ^9^ ^]^ The refinement results are summarized in Table S1 (Supporting Information). The lattice constants of CSO:Tb were expanded as function of *x*, whereas CSO:Tb/Al showed lattice contraction compared with that of CSO:Tb at *x* = 0.010. These results are consistent with the peak shifting observed with XRD.

We investigated the morphology of CSO:Tb and CSO:Tb/Al using scanning electron microscope (SEM). The SEM images of CSO:Tb at *x* = 0.00, 0.01, and 0.02 are shown in Figure 1d,e, respectively. The grains of CSO:Tb were round in shape, and their sizes were reduced as *x* increased. Interestingly, the grain size of CSO:Tb/Al showed larger grain radius compared with that of CSO:Tb (Figure 1f). For a quantitative analysis, we conducted a grain segmentation analysis of the SEM images of CSO:Tb and CSO:Tb/Al using Matlab‐based analysis software. As shown in Figure S1 (Supporting Information), the average grain radii of CSO:Tb at *x* = 0.00, 0.01, and 0.02 were 1.37, 0.47, and 0.46 µm, respectively. The decrease in grain size of CSO:Tb resulted from the ability of Tb ions to inhibit grain boundary mobility.^[^ ^10^ ^]^ Meanwhile, the average grain radius of CSO:Tb/Al was determined to be 0.81 µm. The Al dopant could attribute to grain growth by reduction of melt surface tension during the sintering process.^[^ ^11^ ^]^

The energy‐dispersive X‐ray spectroscopy (EDS) spectra of CSO:Tb at *x* = 0.02 are shown in Figure 1h. The EDS spectra of CSO:Tb exhibited strong multiple peaks at 0.49, 3.43, and 3.67 keV corresponding to O Kα, Sn Lα, and Ca Kα, respectively.^[^ ^12^ ^]^ Furthermore, tiny peaks originating from the Tb ion were observed at 1.20, 6.25, and 6.99 keV, which corresponded to Tb M, Tb Lα, and Tb Lβ_1_, respectively.^[^ ^13^ ^]^ Elemental mapping was employed to obtain the distributions of Ca, Sn, O, and Tb in the sample. According to the elemental mapping images shown in Figure S1a–d (Supporting Information), all the elements were homogeneously distributed in the grains.

### 2.2 Photoluminescence Properties: PL, PLE, and PLQY

**Figure 2:**
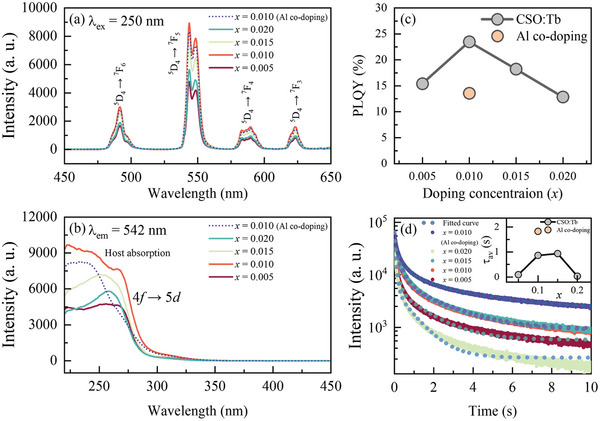
Photoluminescence a) emission (λ_ex_ = 250 nm) and b) excitation (λ_em_ = 542 nm) spectra of CSO:Tb and CSO:Tb/Al. c) Photoluminescence quantum yield of CSO:Tb and CSO:Tb/Al. d) Time‐resolved photoluminescence of CSO:Tb and CSO:Tb/Al.

We explored the photoluminescence (PL) properties of CSO:Tb and CSO:Tb/Al. The PL spectra were obtained by photoexcitation at λ_ex_ = 250 nm. As shown in **Figure** **2**a, sharp PL peaks of Tb^3+^ near 492, 542, 590, and 623 nm were assigned to transitions from ^5^D_4_ to ^7^F_6_, ^7^F_5_, ^7^F_4_, and ^7^F_3_, respectively.^[^ ^14^ ^]^ The photoluminescence excitation (PLE) spectra were measured by monitoring the PL intensity at λ_em_ = 542 nm, which corresponds to the ^5^D_4_ → ^7^F_5_ transition of the Tb^3+^ ions. As shown in Figure 2b, the PLE spectra of CSO:Tb and CSO:Tb/Al exhibited a broad peak at ≈200–250 nm, which corresponds to the combination of the host absorption and the 4*f* → 5*d* transition of Tb^3+^.^[^ ^15^ ^]^ The *f*–*f* transition absorption lines of Tb^3+^ at 300–400 nm were barely observed.^[^ ^16^ ^]^

As shown in Figure 2a, the PL spectra exhibited the highest intensity at *x* = 0.01 (quenching concentration). The quenching concentration of phosphor could be used to determine the energy transfer mechanism.^[^ ^17^ ^]^ The energy transfer between RE ions induced by cross‐relaxation can be used to explain the concentration quenching process. The distance between the ions has a significant impact on the cross‐relaxation of RE ions.^[^ ^17^ ^]^ The determination of the critical distance (R_c_) between RE ions within the host material can be achieved through the Blasse equation, which is expressed as follows^[^ ^17a^ ^]^:

(1) $$R_{c}=\;2{\left[\frac{3V}{4\pi x_{c}Z}\right]}^{1/3}\;$$

where *V* is the unit cell volume, *x*_c_ is the critical concentration for quenching, *Z* is the number of cationic sites in the unit cell, and *x*_c_ is 0.01. The *R*_c_ value was estimated to be 28.66 Å. According to the criterion that *R*_c_ < 5 Å in exchange interactions and *R*_c_ > 5 Å in multipolar interactions, the fact that the *R*_c_ of our samples exceeds 5 Å suggests that the energy transfer mechanism between Tb^3+^ ions in CSO:Tb is likely associated with a multipolar interaction.

To obtain an insight into the luminescent properties of our samples, we also measured the photoluminescence quantum yield (PLQY), which is a measure of the efficiency of a material or substance to convert absorbed light into emitted light. It is defined as the ratio of the number of photons emitted by a material or substance to the number of photons absorbed by that material or substance. The calculated PLQY values of CSO:Tb (λ_ex_ = 250 nm) are shown in Figure 2c. The PLQY had a maximum of 25% at *x* = 0.01 of CSO:Tb. This concentration dependence of PLQY was in good agreement with that of PL intensity, which indicates that the PLQY is an essential factor for determining the PL intensity in our samples. Meanwhile, the PLQY value of CSO:Tb/Al was smaller than that of CSO:Tb. It is worth noting that the Al^3+^ doping could be attributed to the formation of the Ca^2+^ vacancy of [V_Ca_]″ due to the difference in the valence state between Ca^2+^ and Al^3+^. Specifically, two trivalent ions can replace three divalent cations, resulting in the creation of a cation vacancy. These vacancies in the material can give rise to non‐radiative processes that cause the suppression of radiative transitions. Therefore, we suggest that Al^3+^ co‐doping was responsible for the decrease in PLQY by forming Ca^2+^ vacancies in CSO:Tb/Al.

### 2.3 Time‐Resolved Photoluminescence and LPL of CSO:Tb

Now we discuss the luminescence decays of our samples, which is essential for realizing the synaptic behavior. To investigate the luminescence dynamics of CSO:Tb and CSO:Tb/Al, we measured the PL decay curves of the ^5^D_4_ → ^7^F_5_ transition in Tb^3+^ using time‐resolved photoluminescence (TRPL). As shown in Figure 2d, the decay curves of CSO:Tb and CSO:Tb/Al showed very long tails, indicative of the distinct defect related transition processes. Clearly the PL intensities of our samples exhibited an exponential decay as a function of time. We note that CaSnO_3_ has a single crystallographic site for the activator ions, which is expected to have single exponential luminescence decay. However, the multi‐exponential decay behavior can arise from energy transfer processes between activator ions and intrinsic defects of CaSnO_3_, even in a single‐site host.^[^ ^4^, ^18^ ^]^ In our cases, the decay curves were well‐fitted with a three‐component exponential function, which can be expressed as follows^[^ ^7^ ^]^:

(2) $$I\;\left(t\right)=\;y_{0}+A_{1}\exp\left(-\frac{t}{\tau_{1}}\right)+A_{2}\exp\left(-\frac{t}{\tau_{2}}\right)+A_{3}\exp\left(-\frac{t}{\tau_{3}}\right)$$

where I(t), y_0_, A_i_, and τ_i_ correspond to the emission intensities at t, offset intensity, intensities at the *i‐*th components at t = 0, and lifetime constants. The detailed parameters from the fitting are summarized in Table S2 (Supporting Information). From these results, we calculated the average decay time (τ_avg_) using the following equation^[^ ^7^ ^]^:

(3) $$\tau_{avg}=\;\frac{A_{1}{\tau_{1}}^{2}+A_{2}{\tau_{2}}^{2}+A_{3}{\tau_{3}}^{2}}{A_{1}\tau_{1}+A_{2}\tau_{2}+A_{3}\tau_{3}}$$

As shown in the inset plot of Figure 2d, the calculated τ_avg_ of CSO:Tb increased from 93.08 to 914.09 ms, which increased with *x* from *x* = 0.01 to 0.015 and then decreased at *x* = 0.02. The increase in τ_avg_ suggests that the electron/hole trapping and thermally activated process of CSO:Tb predominated as *x* increased up to *x* = 0.15. The decrease in τ_avg_ at *x* = 0.02 is associated with the cross relaxation and the related concentration quenching. Interestingly, we found that the τ_avg_ of CSO:Tb/Al (τ_avg_ = 1821 ms) was much longer than that of CSO:Tb, (τ_avg_ = 860.53 ms) for *x* = 0.01. This finding indicates that the additional defect states formed by co‐doping of Al^3+^ could lead to an increase in τ_avg_.^[^ ^19^ ^]^ We note that the valence state difference between the trivalent Tb^3+^/Al^3+^ and divalent Ca^2+^ ion generates defects such as cation vacancies for the charge compensations. The defect states inhabited within the bandgap can create electron/hole trapping and a thermally activated process that facilitate persistent luminescence.^[^ ^15^, ^20^ ^]^

To confidently observe the LPL property of CSO:Tb and CSO:Tb/Al, we observed afterglow curves following 5 min of UV‐excitation (λ_ex_ = 254 nm).^[^ ^21^ ^]^ As shown in Figure S2 (Supporting Information), prolonged afterglows were observed. Similar to the results from TRPL, the longest afterglow was shown at the *x* = 0.010 of the CSO:Tb series. Furthermore, the CSO:Tb/Al showed a surpassing afterglow compared to the CSO:Tb at *x* = 0.010.

### 2.4 Thermoluminescence

**Figure 3:**
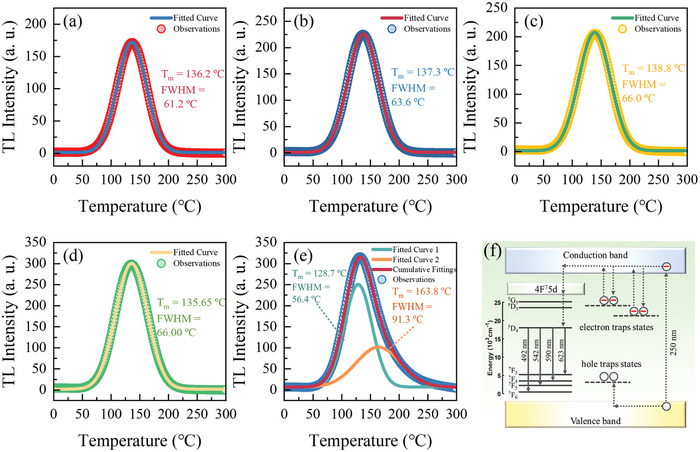
Thermoluminescence in the range of 0–300 °C under β‐ray irradiation (100 Gy) of CSO:Tb at *x* = a) 0.005, b) 0.010, c) 0.015, d) 0.020, and e) CSO:Tb/Al, respectively. The colored solid curves represent the fitted curves. f) Energy diagram of CSO:Tb/Al.

To obtain the detailed information of defect states in the samples, we measured the Thermoluminescence (TL) spectra of CSO:Tb and CSO:Tb/Al with β‐ray irradiation. As shown in **Figure** **3**a–d, the TL spectra commonly showed broad peaks in all samples near T = 130 °C. From the Gaussian peak fitting on the peaks, we calculated the specific trap depths of CSO:Tb by using the following equation^[^ ^22^ ^]^:

(4) $$E_{T}=\left[2.52+10.2\left(\mu_{g}-0.42\right)\right]\;\left(\frac{\kappa T_{m}^{2}}{\omega }\right)-2\kappa T_{m}$$

where T_m_, ε, δ, and κ denote the temperature with Kelvin at maximum intensity of the TL peaks, half‐width low‐ and high‐temperature, and Boltzmann's constant, respectively. Additionally, ω is defined as ω = ε + δ, and µg denotes the asymmetry factor expressed as µg = δ/ω. The fitting results are summarized in Table S3 (Supporting Information). From this, the trap depth energies of CSO:Tb were calculated as 1.073, 1.087, 1.104, and 1.094 eV at *x* = 0.05, 0.10, 0.15, and 0.20, respectively, showing no significant doping dependence.

Interestingly, the TL peak shape of CSO:Tb/Al differed from that of CSO:Tb at *x* = 0.01, which was composed of two peaks (Figure 3f). The TL peak of CSO:Tb/Al can be fitted with two Gaussians positioned at 128.7 and 163.8 °C. The two TL peak structure indicates that additional defect states were introduced by Al^3+^ co‐doping. These results are quite consistent with the PLQY and TRPL of CSO:Tb/Al, that is, the Al co‐doping produces additional defect states to CSO:Tb/Al. The trap depths were calculated to be 1.033 and 1.284 eV.

The luminescent process in our samples is depicted in Figure 3c. The electron/hole trap states revealed by the TL spectra are included. The intrinsic defect states within the material can effectively capture and subsequently release excited electrons. Excited electrons stimulated by UV irradiation undergo diverse relaxation paths. Some of them participate in radiative transitions between the energy levels of Tb^3+^, while others are trapped in the defect states. The long‐running release of the trapped electrons are closely related to the LPL behavior.

### 2.5 Synaptic Behavior 1: PPF

**Figure 4:**
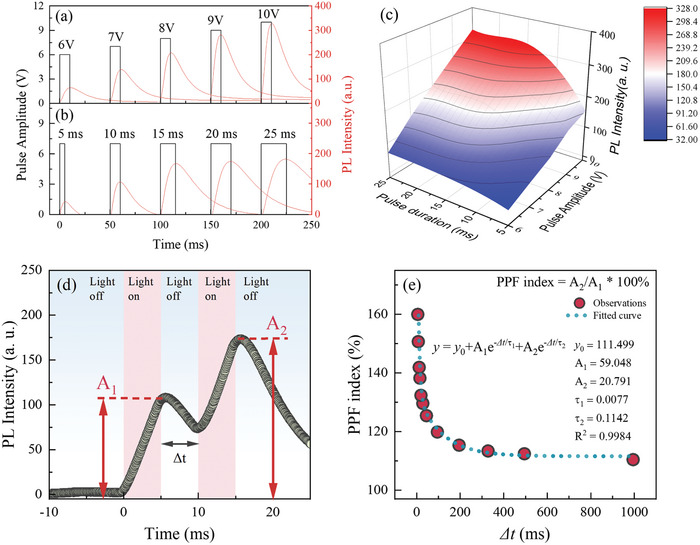
The introduced UV‐pulses (black solid line) and observed PL intensities (red solid lines) with varying pulse a) amplitude and b) duration. PL intensity mapping with varying amplitude and duration of introduced UV‐pulse from CSO:Tb/Al. d) The pair‐pulse facilitation (PPF) behaviors were stimulated by UV‐ pulses with 5 ms of *Δt*, and 5 ms pulse width. e) PPF index variates with pulse time interval *Δt* varied from 5 to 995 ms, and the fitting curves.

Now we discuss the application of the LPL behavior in our samples to the synaptic device. To obtain the validity of CSO:Tb/Al as an artificial synapse, first we investigated PL responses by single pulse excitation and the PPF in luminescent signals of CSO:Tb/Al, which are essential factors in synaptic transmission.^[^ ^1^, ^23^ ^]^ We note that the key requirements of neuromorphic applications include the transition between multiple states in an analog fashion, similar to how neurons integrate incoming signals.^[^ ^24^ ^]^ **Figure** **4**a–c shows the PL intensities from CSO:Tb/Al with varying pulse amplitude and duration. The pulse amplitudes in these observations represent the voltages of the introduced pulse signal applied to the integrated UV‐LED of our sample. The observed PL intensities showed a more non‐linear increase as a function of pulse duration, while exhibiting a linear increase relative to pulse amplitude relatively (Figure 4c). These results indicate that the PL responses could be modulated depending on the introduced pulse conditions. We note that this characteristic is a highly desirable for neuromorphic applications.

Next, we demonstrate that our samples show PPF with the luminescent signals. When the stimulus pulse is introduced, neurotransmitters are produced for a short period of time in biological synapses. The residual neurotransmitters from the first stimulus pulse can enhance the release of neurotransmitters in response to the second pulse. In this regard, PPF refers to an increase in the strength of synaptic transmission in response to two close action potentials. This phenomenon is typically characterized by an enhanced post‐synaptic response to the second of two closely timed stimuli compared with the response to the first stimulus.

For these experiments, we used square waved UV‐light pulses as an excitation spike and recorded the PL intensities as post‐synaptic responses. When two UV‐light pulses with some time delay were introduced to CSO:Tb/Al, the emission intensity curves of CSO:Tb showed two peaks, corresponding to two pulse times. Interestingly, we found that the intensity of the second peak increased significantly compared with the first one (Figure 4d). The PPF index is defined as A_2_/A_1_ * 100%, where A_1_ and A_2_ are the first and second PL response intensities caused by serial input stimulus, respectively. The PPF indexes of CSO:Tb/Al were recorded manipulating the pulse intervals (*Δt*). For clarity, the period of the square wave can be expressed as 1/*f* = *w* + *Δt*, where *f* is the frequency, *w* is the pulse width, and *Δt* is the pulse interval. Therefore, we modified the duty cycle of each frequency to consistently maintain *w* with 5 ms. As shown in Figure 4e, we found that the PPF index reached a remarkable value of 160% at *Δt* = 5 ms. As *Δt* increased, the PPF index decreased and approached 113% at *Δt* = 995 ms. The PPF index in our samples is quite comparable to the performance of several existing optoelectronic devices, which achieve the potentiation index in the range of 139% to 165% at *Δt* = 100–500 ms.^[^ ^25^ ^]^ Furthermore, the characteristic curves of the PPF index as a function of *Δt* can be fitted to a biexponential function. Our fitting results demonstrated a strong match, with an R^2^ value of 0.9984. Interestingly, these changes are quite similar to a typical behavior found in biological synapses.^[^ ^26^ ^]^

### 2.6 Synaptic Behavior 2: Spike‐Number Dependent Potentiation

**Figure 5:**
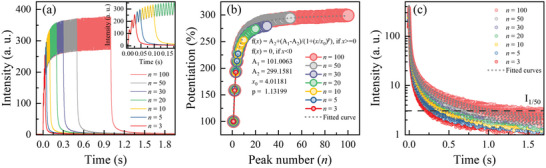
Pulse‐number dependent synaptic functionalities of CSO:Tb/Al with pulse number varying from 3 to 100. a) Observed luminescence intensities by serial pulses with *n* = 3–100. b) Potentiation by serial pulses with *n* = 3–100. c) Short‐term potentiation (STP) to long‐term potentiation (LTP) transition by serial pulses with *n* = 3–100.

As another key feature of synaptic behaviors, we investigated the impact of pulse number on the potentiation of luminescent signals in CSO:Tb/Al. The potentiation was calculated using the formula *I_n_*/*I_0_* * 100%, where *I_0_* represents the emission intensity from the first pulse, and *I_n_* represents the emission intensity from *n‐*th pulse. The pulse number (*n*) changed in range from *n* = 3 to *n* = 100. *w* and *Δt* were fixed at 5 ms. The exact configurations of the pulse sequences are depicted in Figure S3 (Supporting Information). As shown in **Figure** **5**a, the intensity of PL peaks exhibited a progressive increase as *n* increased. Notably, a potentiation of 300% was achieved for *n* = 100 (Figure 5b). We repeated the experiments and obtained the same potentiation value for a specific *n*, which demonstrates remarkable consistency and repeatability. It is worthwhile to mention that these observed changes closely resemble a phenomenon observed in biological synapses. In biological synapses, an increase of neurotransmitters (i.e., the influx of ions such as Na^+^, K^+^, and Ca^2+^) leads to increases of sensitivity in the receptors of neurons.^[^ ^1^ ^]^ Interestingly, the dependence of potentiation increments on *n* exhibited a nonlinear curve that could be modeled with a logistic function. It is worth emphasizing that the logistic function holds a well‐accepted role as an activation function in NNs.^[^ ^27^ ^]^ As shown in Figure 5b, the application of the logistic function to the potentiation increments at *n* = 100 showed a notable goodness‐of‐fit with an R^2^ value of 0.999.

Synaptic plasticity, a fundamental aspect of neural communication, can be classified into Short‐Term Potentiation (STP) and Long‐Term Potentiation (LTP).^[^ ^28^, ^29^ ^]^ STP manifests when a stimulus triggers a surge of ion flux at the presynaptic membrane, prompting neurotransmitter release and bolstering synaptic activity. However, the effects of STP are short‐lived, typically spanning mere milliseconds, as ion flux is swiftly expelled from the postsynaptic membrane. With repeated stimulation, synaptic efficacy can be prolonged, a phenomenon known as LTP. This transition from STP to LTP involves structural modifications in the synaptic junction and alterations in dendritic size, driven by the dynamics of ion flux. Thus, the interplay between STP and LTP regulates the adaptability and enduring nature of biological synapses.^[^ ^28^, ^29^ ^]^ It is noted that within synaptic devices, STP can be gradually transformed into LTP by external stimulus.^[^ ^29^ ^]^ The transition from STP to LTP holds crucial significance as a fundamental characteristic in synaptic devices because it enables retaining of the state or configuration over an extended period by repetitive stimulations.^[^ ^29^ ^]^

Interestingly, CSO:Tb/Al showed the STP to LTP transition under spike‐number variations. To verify the transition, the response signal should be measured with respect to the time duration to specific thresholds.^[^ ^30^ ^]^ As shown in Figure 5c, for a given threshold value (2% of the first luminescence peak intensity, denoted as I_2%_), as the value of *n* increases, the time taken to reach I_2%_ (t_2%_) increases: t_2%_ = 358 ms for *n* = 3 and t_2%_ = 1836 ms for *n* = 100. The increase in t_2%_ partly results from the higher intensity at the last pulse with increasing *n*. To obtain deeper insight into the STP to LTP transition, the PL decay curves were analyzed based on exponential curve fitting. We utilized Equation (2) for fitting. The detailed fitting results, including t_2%_, are summarized in Table S4 (Supporting Information). We denoted three terms with the fast (A_1_ and τ_1_), intermediate (A_2_ and τ_2_), and slow (A_3_ and τ_3_) components. Interestingly, A*_i_* showed systematic changes with the increase in *n*. As *n* increased, the fast component (A_1_) decreased, whereas the intermediate and slow components (A_2_, and A_3_) increased. We attributed the observed changes to the increase in electron trapping within deeper defect states as *n* increases. Meanwhile, the corresponding time constants τ*_i_* decreased slightly as *n* increased. Consequently, the τ_avg_ increased as *n* increased. We attribute the increase in t_2%_ with *n* to both the increase in the last peak intensity and increases of slower components in the decay process. It is important to note that the decay time of LPL is very versatile, depending on the combinations between RE ions and host materials. Consequently, the transition from STP to LTP could be more successfully demonstrated with deep optimization in further research.

### 2.7 Synaptic Behavior 3: Spike‐Timing Dependent Potentiation

**Figure 6:**
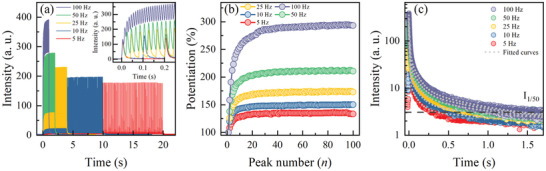
Pulse‐timing dependent synaptic functionalities of CSO:Tb/Al with 100 serial pulses (*n* = 100) and different frequencies (*f*) from 5 to 100 Hz. a) Observed luminescence intensities at *f* = 5–100 Hz. b) Potentiation by *n* = 100 at *f* = 5–100 Hz. c) STP to LTP transition at *f* = 5–100 Hz.

Next, we investigated the impact of pulse frequency (*f*) on potentiation in CSO:Tb/Al with the variation of *f* ranging from 5 to 100 Hz (i.e., *Δt* = 5–195 ms). *w* and *n* were fixed at 5 ms and *n* = 100, respectively. The exact configurations of the pulse sequences are depicted in Figure S4 (Supporting Information). It is noteworthy that, as shown in **Figure** **6**a,b, the intensities of the final peaks at *n* = 100 increased with increasing *f*. The potentiation at *n* = 100 increased from 140% to 300% as *f* increased from 5 to 100 Hz.

Similar to the case of the spike‐number dependent potentiation, we observed the STP to LTP transitions under spike‐timing variations: t_2%_ = 696.4 ms for *f* = 5 Hz and t_2%_ = 1836 ms for *f* = 100 Hz. The transition from STP to LTP was quantified by fitting with Equation (2) on PL decay curves. The detailed fitting parameters were acquired through exponential decay curve fitting to quantify the STP to LTP transition (Table S4, Supporting Information). Interestingly, as shown in Figure 6c, the dependence of A*_i_* and τ*_i_* on *f* showed trends similar to the pulse‐number dependent results: as *f* increased, the fastest component (A_1_) decreased, while longer components (A_2_, and A_3_) increased. Meanwhile, τ*_i_* decreased as the frequency increased. As the decrease in τ*_i_* is predominant compared with the changes in A_i_, τ_avg_ decreased as the frequency increased. However, the observed t_2%_ was longer at higher *f* owing to the stronger intensity of the last peak. This occurs because the changes in t_2%_ with *f* can be attributed more to the alteration of the last peak intensity than to changes in the components of the decay process.

### 2.8 Synaptic Behavior 4: Neural Network for Handwritten Digit Recognition

Now, we demonstrate that our luminescent synaptic behavior could be employed in an NN for various applications. For example, we consider the recognition of characters from handwritten images, which poses a significant challenge within the realm of optical character recognition.^[^ ^31^ ^]^ Indeed, the text extracting from real images is an arduous task owing to the huge variations in font size and shape, texture, background, etc. Among the available techniques, NNs have emerged as a highly effective tools for image recognition.^[^ ^32^ ^]^ Therefore, the MNIST handwritten digit recognition is one of the most fundamental tests for assessing how well the artificial synaptic device works.

**Figure 7:**
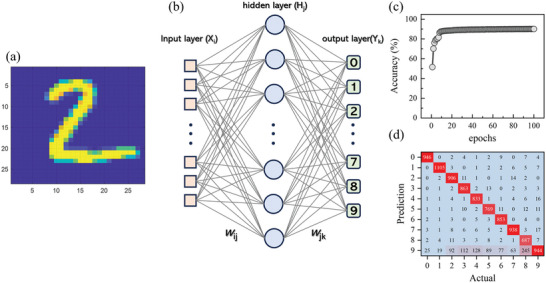
a) A handwritten digit from the MNIST dataset composed of 28 by 28 pixels. b) Schematic of a neural network (NN) for handwriting recognition. c) Recognition accuracy of the NN using the activation function from CSO:Tb/Al, as a function of epochs. d) Confusion matrix of the NN using the activation function from CSO:Tb/Al after 100 epochs.

Using the luminescent synaptic behavior of CSO:Tb/Al, we developed an NN for recognizing hand‐written digits. The NN was developed using Matlab codes and including weight updating by back propagations. We integrated the pulse‐number dependent potentiation curve of CSO:Tb/Al (Figure 5b) as the activation function into the NN. The activation functions in NNs, which correspond to the characteristic of the interconnection among the neurons, can have arbitrary scale owing to the weight updating during training. We calculated the linearity of pulse number‐dependent potentiation to be 13.13 (Figure S5, Supporting Information).^[^ ^25a^ ^]^ Our approach involved a dataset of 60 000 MNIST handwritten samples for training purposes, and a separate set of 10 000 samples was utilized for validation (**Figure** **7**a).^[^ ^33^ ^]^ In our NN architecture, a hidden layer comprising 100 nodes was implemented. This layer was intricately connected to both the input and output layers, forming a complete linkage as depicted in Figure 7b. As shown in Figure 7c, the accuracy of the NN approaches ≈51% after just one epoch, and it reaches up to 90% after 20 epochs. We note that one epoch corresponds to 60 000 learning phases. Notably, the confusion matrix, which illustrates the intricate comparison of accuracy between the NN predictions and the actual labels, exhibited a distinct diagonal distribution pattern after 100 epochs (Figure 7d). This diagonal distribution indicates that the predictions of the NN are highly accurate. Our results indicate that our LPL system should be remarkably effective as an artificial synapse for the NN simulations. For more clarity on our simulation, we included the weight matrixes and biases from MNIST handwritten digit recognition in the learning processes in Figure S6 (Supporting Information).

We made another image recognition NN, MNIST fashion image recognition, in the same way with the MNIST handwritten digit recognition explained above, as shown in Figure S7 (Supporting Information). We note that MNIST‐fashion recognition refers to the task of classifying images of fashion items into specific categories using machine learning techniques.^[^ ^34^ ^]^ The dataset consists of 60 000 training images and 10 000 testing images, each belonging to one of 10 different classes: T‐shirt/top, Trouser, Pullover, Dress, Coat, Sandal, Shirt, Sneaker, Bag, and Ankle boot. We found that the accuracy of the NN approaches approximately 80% after 75 epochs, and finally, 80.9% of accuracy was achieved after 100 epochs. Notably, the confusion matrix, which illustrates the intricate comparison of accuracy between the NN predictions and the actual labels, exhibited a distinct diagonal distribution pattern after 100 epochs, indicating high performance of our luminescent synaptic samples. For more clarity on our simulation, we included the weight matrixes and biases from MNIST fashion recognition in the learning processes are represented in Figure S8 (Supporting Information).

### 2.9 Synaptic Behavior 5: LPL Driven Reservoir Computing

We demonstrate the second NN simulation with RC layer, by employing the synaptic behaviors of our luminescent samples. It is worthwhile to note that RC is a promising algorithm for temporal data processing. Internal reservoirs convert time‐varying signals to high‐dimensional states. These high‐dimensional states are subsequently processed by a basic readout layer. This mechanism leads to a notable decrease in both the size of the network and the costs associated with training.^[^ ^35^ ^]^ By using the LPL properties of our samples, such as, the serial pulse facilitation, we set up the LPL‐driven RC, which can store and retain four optical pulses, and effectively create a 4‐bit optoelectronic reservoir computing.

**Figure 8:**
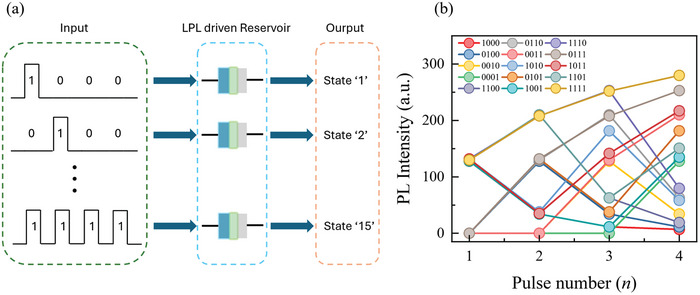
(a) The schematic of the reservoir process and b) the output states of 4‐bit reservoir property using the LPL of CSO:Tb/Al. b) The observed output states of 4‐bit reservoir property using CSO:Tb/Al.

As shown in **Figure** **8**a, the input signals comprise 4 optical pulses representing input bits, spanning from “0001” to “1111”. As these optical pulses are introduced sequentially into the device, the pulse stream yields outputs labeled from ″state 1″ to ″state 15″. The corresponding signals of each state are monitored at the 4th peaks, as shown in Figure 8b. Notably, the 15 reservoir states at 4th pulse exhibited highly unique values, providing clear distinguishability. This RC process is based on one of the representative synaptic behaviors, and can be evidence of the feasibility of our LSD.

**Figure 9:**
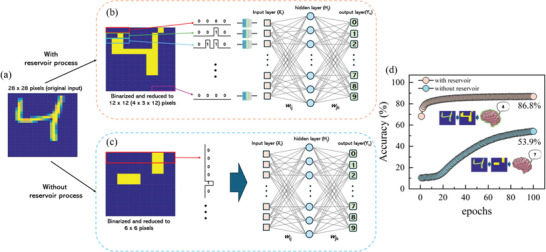
The schematic of LPL driven reservoir process. a) Original input image (28 × 28), b) The sequence of reservoir computing process using the 4‐bit reservoir using CSO:Tb/Al. c) The sequence of general NN process without reservoir. The numbers of nodes in two NNs for input layer, hidden layer, and output layer nodes are 36, 30, and 10, respectively. d) Comparison of recognition accuracy of two NNs as a function of epochs.

By using the obtained reservoir property, we simulated an in‐sensor RC network integrated NN for MNIST handwritten digit recognition (**Figure** **9**a–c). To demonstrate the feasibility of our reservoir, we constructed a NN with 36 × 30 × 10 size. The initial 28 × 28 MNIST images are down sampled to 12 × 12 size, and then every group of 4 pixels within the images can be transformed to one of 15 states. Through these two steps, the initial 28 × 28 images are reduced to 3 × 12 ones, and the number of reservoirs and input neurons are matched (i.e., reservoirs: 3 × 12, input neurons: 36). These states are introduced into the reservoir units, which is connected to the input neuron, as depicted in Figure 9b.

For comparison, we simulated an additional neural network with identical in size but without reservoirs. To match the count of image pixels with the number of input neurons, the input images for this general neural network were downscaled to a 6 × 6 resolution, as depicted in Figure 9c.The results highlighted a significant improvement in the learning process when applying LPL‐driven RC to NN, as depicted in Figure 9d. After 100 epochs, the RC‐aided NN achieved an accuracy of 86.8%, while the NN without RC achieved only 53.9% accuracy. These findings demonstrate that LPL‐driven RC can effectively enhance NN efficiency by reducing the required number of neurons for specific tasks. The obtained weight matrixes and biases from our in‐sensor RC network integrated NN in the learning processes are shown in Figure S9 (Supporting Information).

### 2.10 Perspectives of Synaptic Functionality Based on LPL of RE‐Ions

In the current study, we succeeded in utilizing the LPL property for realizing synaptic devices. In detailed examinations including PPF, pulse‐number/timing dependent potentiation, and NN simulations, we observed the substantial efficiency of the LPL property as an artificial synapse. Notably, key factors contributing to potentiation characteristics, such as, variations in stimulus repetition and timing, have been distinctly identified and analyzed, providing valuable insights into the adaptability and functionality of the LPL‐based artificial synapses.

We elaborate on the differences between our luminescent synaptic device (LSD) and other optoelectronic synaptic devices (OESDs). OESDs using metal‐oxide/organic semiconductor, quantum dot (QD), and 2D materials have many advantages for practical applications owing to their compatibility with common electronic‐based platforms.^[^ ^36^ ^]^ The main mechanisms in operation of OESDs, are photoconductivity and photoelectron effect based on the dynamics of defects (e.g., migration of ion vacancies). With combination of these major properties to memristic transport, there have been significant advances in developing the OESDs.^[^ ^1^ ^]^ For example, indium gallium zin oxide (IGZO) shows synaptic functionalities based on its inherent photoconductivity due to the dynamics of oxygen vacancies.^[^ ^37^ ^]^ As the process of forming or neutralizing ionized oxygen vacancies in IGZO is time‐consuming, its photoconductivity is sufficiently persistent for creating synaptic plasticity. The operation time scale in PPF typically ranges from 1 to 100 s, which is effective for the establishment of long‐term memory, but not valid for a rapid learning process.^[^ ^36^, ^37^ ^]^ Meanwhile, optical transition‐based optoelectronic synaptic materials show a faster process. Zhao et al. reported synaptic functionalities using Si‐ nano crystal light‐emitting diodes within the milli‐second time scale (1–10^4^ ms), which is comparable to the typical decay time of a signal in a biological synapse.^[^ ^36c^ ^]^ These results indicate that optical transition‐based synaptic materials have the potential to create artificial synapses for high‐speed learning processes, but their fast relaxation is less favorable for long‐term memory.

While our LSDs exhibit most of synaptic behaviors of OSEDs, such as, PPF, pulse number/frequency dependent potentiation, STP/LTP, and delicate NN simulations, our LSDs could be made applicable for both fast learning and long‐term memory through the control of the LPL decay time by harnessing the trap states. Additionally, through the highly discernible and various transition paths of *f*–*f* transitions in RE‐ions, these systems could provide significant contrasts in wavelengths between the stimulus and response lights, as well as multi‐channel processing, that is, multi‐wavelength excitation/emission. Furthermore, LSDs are based on the optical input and output through the luminescence. Since light intensity strongly depends on the architecture and structure of devices, LSDs could offer plenty of versatility for optimization and design. Moreover, persistent luminescence can be easily manipulated by controlling various parameters, such as host material, activators, sensitizer, defects, excitation/emission energy, and so on. Lastly, we emphasize that the introduction and recording of light signals are easily accessible due to recent advances in display and camera sensors. The realization of the luminescent synaptic device can be achieved by integrating the samples into 2D LED arrays and a CMOS/CCD sensor. Therefore, we suggest that LSDs with wide tuning range in time and photon energy has significant potential for the development of novel synaptic devices. Our summarized suggestions are as below: 1) The LSD is based on the optical input and output through the luminescence. 2) Since the light intensity depends strongly on the architecture and structure of devices, the LSD could offer a plenty of versatility for optimization and design. 3) The persistent luminescence can be easily manipulated by controlling various parameters, such as host material, activators, sensitizer, defects, excitation/emission energy, and so on. 4) The photonic signal of LOSDs can convert to the electric one, compatible to the current electronic systems.

## 3 Discussion

In this study, we demonstrated the potential applications of CSO:Tb and CSO:Tb/Al as synaptic materials. The emission response, facilitated by the LPL property of Tb^3+^ in CSO:Tb/Al, exhibited significant potentiation under serial UV‐pulse inducement. To validate the synaptic behavior of CSO:Tb/Al, we investigated major synaptic behaviors such as PPF, pulse‐number/timing dependent potentiation, and pulse‐number/timing dependent short‐ to long‐term plasticity transition. The potentiation in PL responses of CSO:Tb/Al showed a clear dependence on pulse number and timing. The PPF index reached 160% at *Δt* = 5 ms, and the potentiation enhancement increased with more pulses and shorter intervals. In both experiments, the STP to LTP transitions were identified. By using the activation function obtained from the pulse‐number dependent potentiation of CSO:Tb/Al, we succeeded in simulating NNs that for two different image recognition tasks which are MNIST handwritten digit recognition and MNIST fashion recognition. We also have showcased the effectiveness of LPL‐driven optical RC capabilities in our samples. These results indicate that the synaptic behavior based on LPL property of RE ions can be a great candidate for synaptic materials.

## 4 Experimental Section

### Materials and Synthesis

Tb^3+^‐doped CaSnO_3_ (CSO:Tb) and Tb^3+^/Al^3+^‐doped CaSnO_3_ (CSO:Tb/Al) samples were synthesized using the solid‐state reaction method.^[^ ^38^ ^]^ High‐purity CaCO_3_, SnO_2_, Tb_2_O_3_, and Al_2_O_3_ were used as raw materials. First, samples were synthesized with the concentration of Tb ions designated to be substituted for Ca ions varying from 0.00 to 0.02. In this way, Ca_(1‐_ *_x_*_)_Tb*_x_* SnO_3_ was prepared for *x* = 0.00–0.02 according to the following mixture rate of raw materials, CaCO_3_: Tb_2_O_3_: SnO_2_ = 1 – *x*: *x*/2: 1. Second, samples were synthesized with the concentration of Tb and Al ions fixed as 0.01, that is, Ca_0.98_Tb_0.01_Al_0.01_SnO_3_. Therefore, the Tb/Al co‐doping sample was prepared with the following predetermined proportions: CaCO_3_: Tb_2_O_3_: Al_2_O_3_: SnO_2_, = 0.98: 0.01/2: 0.01/2: 1. The mixed powders were milled and pressed into cylinder shape pellets, calcined at 900 °C for 12 h, and then sintered at 1400 °C for 20 h.

### Materials Characterization

The XRD patterns of the samples were measured using a Bruker AXS D2 system (Cu target X‐ray tube), and Rietveld refinement was performed for obtaining the detailed structural information of the samples.^[^ ^9^ ^]^ The SEM and EDS images were obtained using a Carl Zeiss Gemini SEM 300 system in the backscattering electron mode. The PL and PLE spectra were acquired using spectrofluorometry (JASCO FP‐8500).^[^ ^39^ ^]^ The photoluminescence quantum yield and decay curves were measured using an integrating sphere (JASCO ILF‐835). The TRPL spectra were measured using a spectrofluorometer (FluoroMax Plus, HORIBA) equipped with a xenon arc lamp. To study the TL responses, the samples were irradiated using a 90Sr/90Y beta source at a dose rate of 0.086 Gy s^−1^. All measurements were performed using an automatic Risø TL/OSL‐DA‐15 reader system. The UV‐pulse induced photo emission responses were recorded using a manually made detection system constructed with a function generator (YOKOGAWA FG300), a commercial UV LED with a 250 nm peak, and a high‐sensitivity light sensor (PASCO CI‐6604). The light sensor opening was covered with a 400 nm filter to block the signal from the excitation light pulses.

## Conflict of Interest

The authors declare no conflict of interest.

## Supporting information

Supporting Information

## Data Availability

The data that support the findings of this study are available from the corresponding author upon reasonable request.
