# Supplementary material for: Optoelectronic Synapse Behaviors in Tb3+ and Al3+ Co‐Doped CaSnO3 with Long‐Persistent Luminescence
Source: Adv Sci (Weinh). 2024 Jun 26;11(32):2402848. doi: 10.1002/advs.202402848 (PMC11348126; doi:10.1002/advs.202402848)
Supplement: Supplementary file 1 — Supporting Information [file ADVS-11-2402848-s001.pdf]

ADVANCED SCIENCE
Open Access

Supporting Information

for *Adv. Sci.*, DOI 10.1002/advs.202402848

Optoelectronic Synapse Behaviors in Tb^{3+} and Al^{3+} Co-Doped $CaSnO_3$ with Long-Persistent Luminescence

Sangwon Wi, Minjae Jeong, Kwanchul Lee and Yunsang Lee**

Optoelectronic Synapse Behaviors in Tb^{3+} and Al^{3+} Co-doped $CaSnO_3$ with Long-persistent Luminescence

Sangwon Wi, Minjae Jeong, Kwanchul Lee, and Yunsang Lee

Department of Physics and Integrative institute of Basic Sciences, Soongsil University, Seoul 06978, Republic of Korea

Supplementary Information

Table S1. Structural parameters obtained from CSO:Tb and CSO:Tb/Al at x = 0.00 – 0.02, using the Rietveld refinement technique.

			$CaSnO_3$ (*Pnma*, 62)			
Tb^{3+} concentration (x)	0.000	0.005	0.010	0.015	0.020	0.010 (Al co-doping)
a (Å)	5.658	5.656	5.658	5.661	5.667	5.661
b (Å)	7.883	7.887	7.890	7.892	7.890	7.897
c (Å)	5.516	5.522	5.523	5.522	5.519	5.521
Cell-volume (Å^3)	246.04	246.33	246.57	246.72	246.77	246.79
Ca(Tb/Al) (4c)						
x	0.0468	0.0489	0.0496	0.0497	0.0530	0.0502
y	0.7500	0.7500	0.7500	0.7500	0.7500	0.7500
z	0.9781	0.9894	0.9862	0.9880	0.9884	0.9869
Sn (4b)						
x	0.0000	0.0000	0.0000	0.0000	0.0000	0.0000
y	0.0000	0.0000	0.0000	0.0000	0.0000	0.0000
z	0.5000	0.5000	0.5000	0.5000	0.5000	0.5000
O (8d)						
x	0.1932	0.1945	0.2231	0.1999	0.1926	0.2079
y	0.0484	0.0395	0.0579	0.0464	0.0460	0.0599
z	0.2046	0.1879	0.2068	0.1929	0.1876	0.2046
O (4c)						
x	0.0347	0.0353	0.0433	0.0428	0.0322	0.0421
y	0.2500	0.2500	0.2500	0.2500	0.2500	0.2500
z	0.6196	0.6245	0.5983	0.6129	0.6016	0.5975
R_{wp}	14.73	7.30	12.35	13.40	18.75	12.03
χ^2	1.65	1.12	1.31	1.82	1.98	1.70

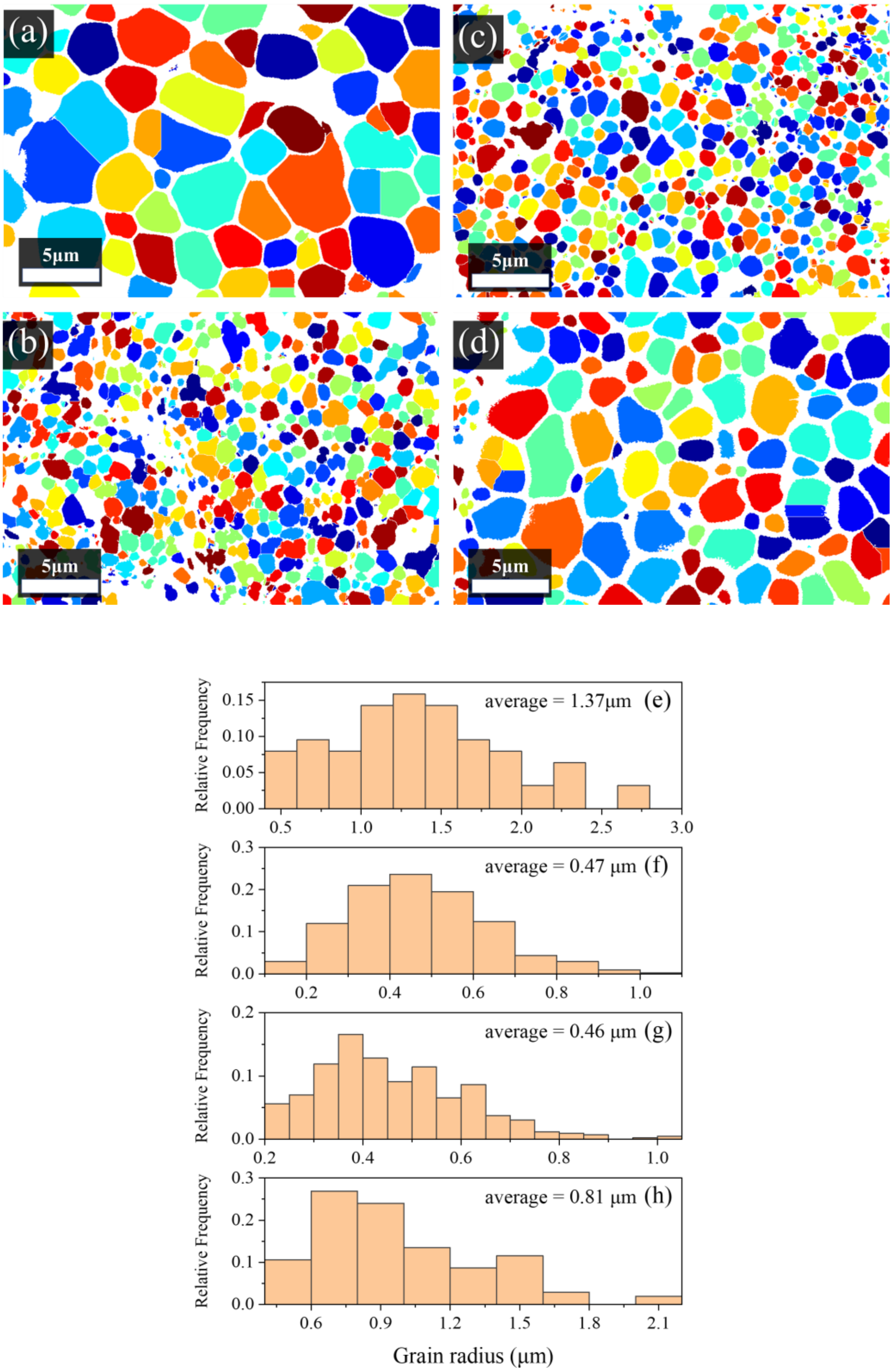

Figure S1. Grain segmentation map of CSO:Tb at x = (a) 0.00, (b) 0.010, (c) 0.020, and (d) CSO:Tb/Al, respectively. The colors were assigned as randomly for identifying the segmentation. (e) – (h) representing the histograms of grain radius from the segmentation maps of (a) – (d), respectively.

Table S2. Fitting parameters for the exponential decay curves of CSO:Tb/Al using TRPL. The values in brackets indicate relative weights.

	Fitting parameters								
Tb^{3+} concentration (*x*)	A_1	A_2	A_3	τ_1 (ms)	τ_2 (ms)	τ_3 (ms)	τ_{avg} (ms)	y_0	R^2
0.005	6425003 (99.26%)	42826 (0.66%)	5061 (0.08%)	19.55	142.93	1375.74	93.08	572.31	0.998
0.010	5592528 (86.40%)	725610 (11.21%)	154752 (2.39%)	46.22	265.32	1942.14	860.53	870.20	0.999
0.015	5072122 (78.36%)	1158191 (17.89%)	242577 (3.75%)	64.94	317.88	1993.49	933.37	914.09	0.9997
0.020	6445458 (99.58%)	25168 (0.39%)	2264 (0.03%)	14.02	99.84	874.42	34.25	261.82	0.998
0.010 (Al co-doping)	4391341 (67.84%)	1565161 (24.18%)	516388 (7.98%)	74.73	393.56	2819.07	1821.24	2304.08	0.9996

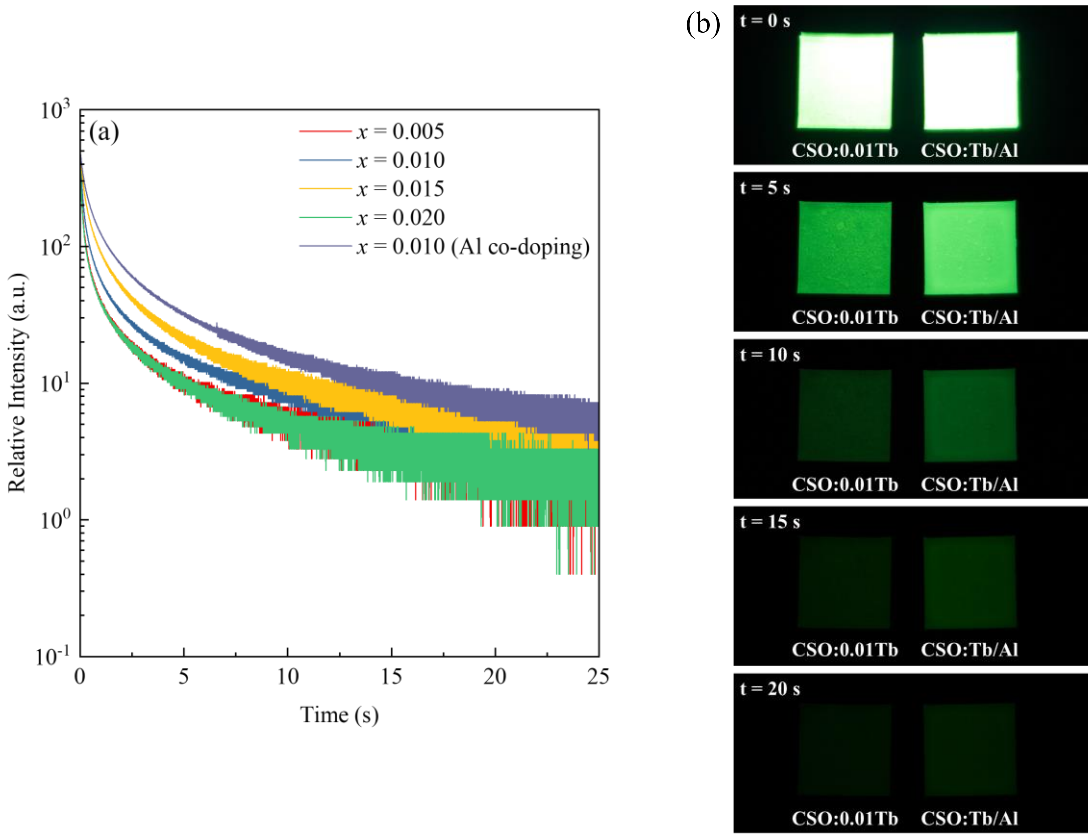

Figure S2. (a) Afterglows after 5 min of UV-light irradiation (λ_{ex} = 254 nm) of CSO:Tb and CSO:Tb/Al. (b) Photographs of afterglows after 5 min of UV-light irradiation with CSO:0.01Tb and CSO:Tb/Al.

Table S3. Fitting parameters of Gaussian fitting to the TL peaks of CSO:Tb and CSO:Tb/Al.

	Fitting parameters					
Tb^{3+} concentration (x)	T_m (°C)	FWHM (K)	ε (K)	δ (K)	Trap depth (eV)	R^2
0.005	136.2	61.2	378.7	439.9	1.073	0.999
0.010	137.3	63.6	378.7	442.3	1.087	0.999
0.015	138.8	66.0	378.7	444.7	1.104	0.999
0.020	135.65	66.0	376.3	442.3	1.094	0.999
0.010 (Al co-doping) #1	128.7	56.4	373.9	430.3	1.033	0.999
0.010 (Al co-doping) #2	163.8	91.3	391.2	482.5	1.284	0.999

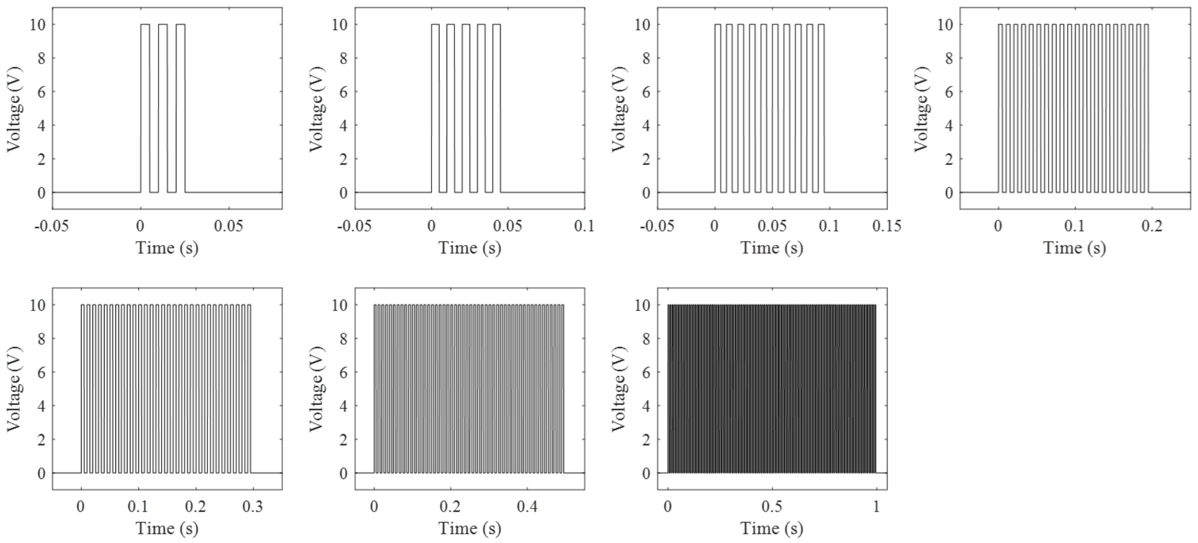

Figure S3. Pulse sequences of (a) n = 3, (b) 5, (c) 10, (d) 20, (e) 30, (f) 50, and (g) 100, used in pulse number dependent potentiation of CSO:Tb/Al, respectively.

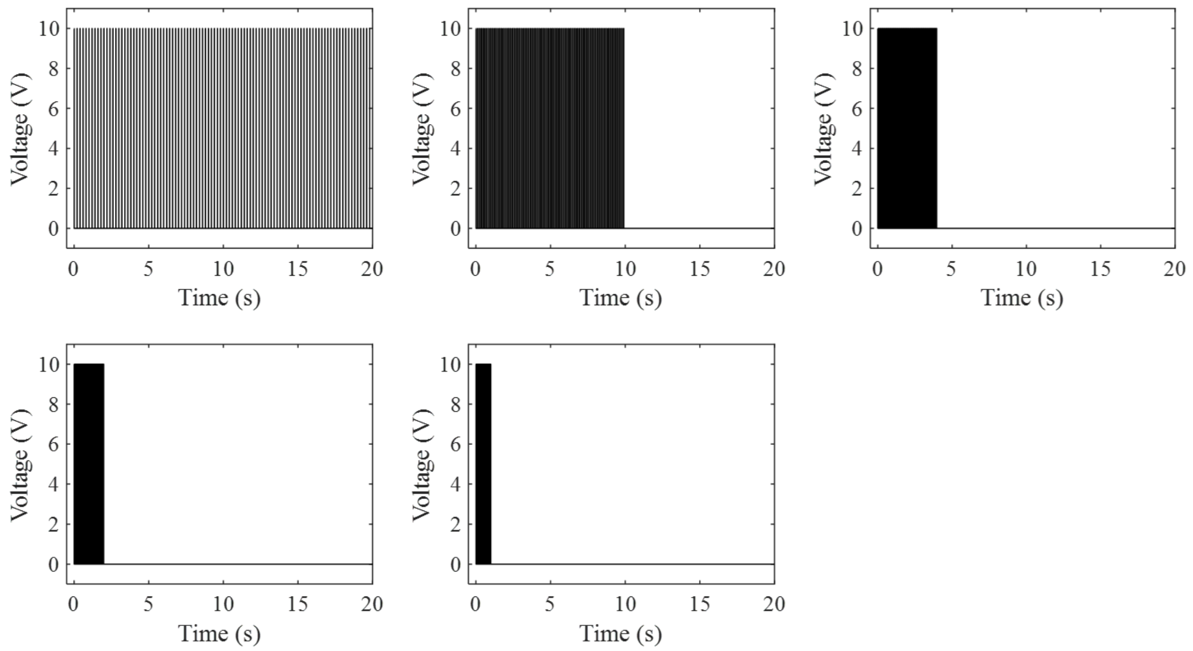

Figure S4. Pulse sequences of (a) f = 5 Hz, (b) 10 Hz, (c) 25 Hz, (d) 50 Hz and (e) 100 Hz, used in pulse timing dependent potentiation of CSO:Tb/Al, respectively.

Table S4. Fitting parameters for the exponential decay curves of CSO:Tb/Al depend on pulse sequences. The values in brackets indicate relative weights.

Pulse sequence		Fitting parameters									
Pulse number (n)	Frequency (Hz)	A_1	A_2	A_3	τ_1 (ms)	τ_2 (ms)	τ_3 (ms)	τ_{avg} (ms)	y_0	R^2	$t_{2\%}$ (ms)
3	100	269.38 (94.44%)	13.87 (4.86%)	2.00 (0.70%)	7.77	71.75	515.97	150.51	1.51	0.997	358.0
5	100	303.87 (92.52%)	21.19 (6.45%)	3.39 (1.03%)	7.93	67.86	473.16	160.50	1.61	0.997	582.3
10	100	338.35 (91.03%)	28.53 (7.67%)	4.80 (1.29%)	8.05	65.79	453.75	167.31	1.71	0.998	763.1
20	100	348.50 (88.97%)	36.13 (9.22%)	7.09 (1.81%)	7.90	62.76	425.13	179.91	1.86	0.998	962.4
30	100	355.23 (88.68%)	37.30 (9.31%)	8.04 (2.01%)	8.02	64.94	425.52	188.16	2.00	0.998	1101
50	100	358.39 (88.03%)	38.83 (9.54%)	9.92 (2.44%)	8.01	65.52	422.60	204.15	2.13	0.998	1258
100	100	358.41 (87.39%)	40.52 (9.88%)	11.22 (2.74%)	7.86	62.28	387.01	340.17	2.43	0.998	1836
100	5	183.62 (94.06%)	9.87 (5.05%)	1.74 (0.89%)	7.77	101.40	1399.73	723.62	1.66	0.993	696.4
100	10	199.17 (92.58%)	13.28 (6.17%)	2.68 (1.24%)	7.70	94.32	1063.45	560.65	1.82	0.994	1308
100	25	227.68 (90.20%)	19.85 (7.87%)	4.88 (1.93%)	7.91	83.52	722.01	386.07	2.02	0.997	1538
100	50	268.00 (88.33%)	28.83 (9.50%)	6.58 (2.17%)	7.98	79.46	688.64	370.45	2.06	0.998	1723
100	100	358.41 (87.39%)	40.52 (9.88%)	11.22 (2.74%)	7.86	62.28	387.01	340.17	2.43	0.998	1836

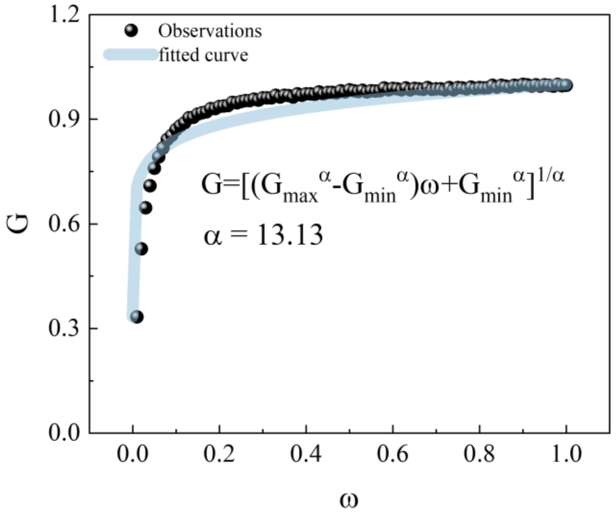

Figure S5. The fitting curve for linearity of pulse number dependent potentiation of CSO:Tb/Al. α represents the linearity, ω ranges from 0 to 1 representing the entire pulse cycle (0 at the beginning of the cycle and 1 at the end). G denotes the observed emission intensity, while G_{min} and G_{max} correspond to the minimum and maximum responses, respectively.

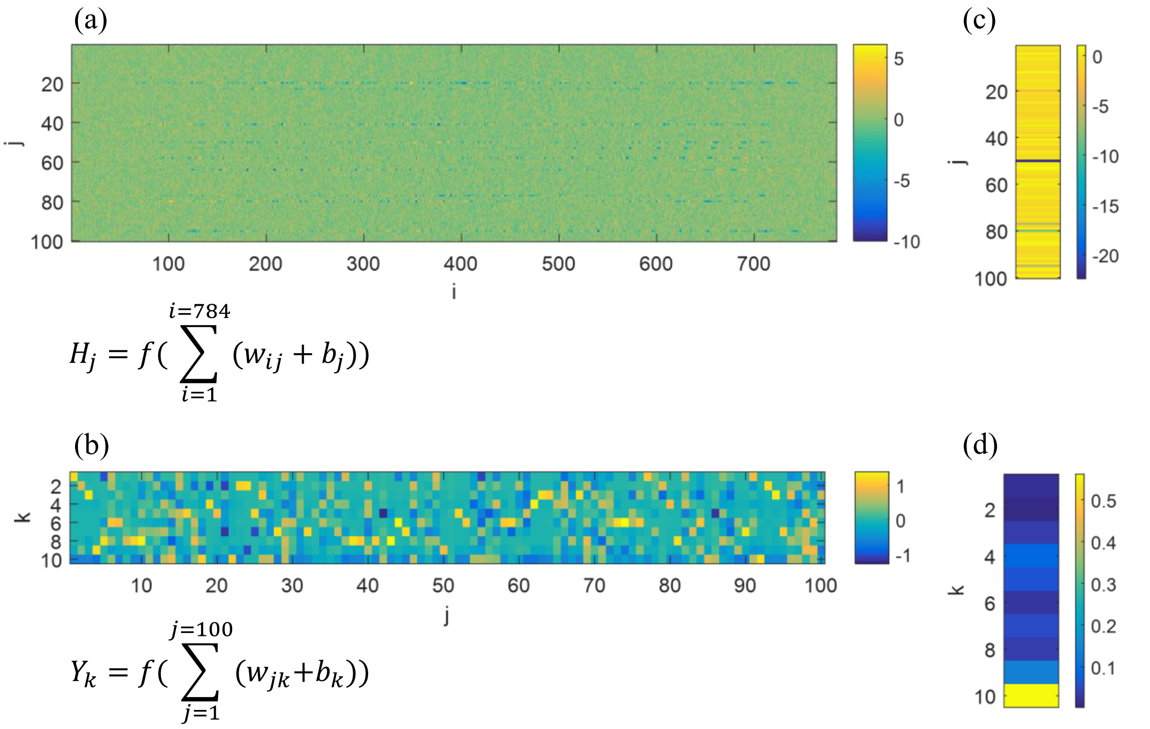

$$H_j = f(\sum_{i=1}^{i=784} (w_{ij} + b_j))$$

$$Y_k = f(\sum_{j=1}^{j=100} (w_{jk}+b_k))$$

Figure S6. The weight matrix of the connection (a) between input layer and hidden layer (w_{ij}), and (b) between hidden layer and output layer (w_{jk}). The bias of the connection (c) between input layer and hidden layer (b_j), and (d) between hidden layer and output layer (b_k) from NN for MNIST handwritten digits recognition with 784×100×10 nodes, after 100 epochs. The expressions for H_j and Y_k are representing the signal propagation in processing of NN.

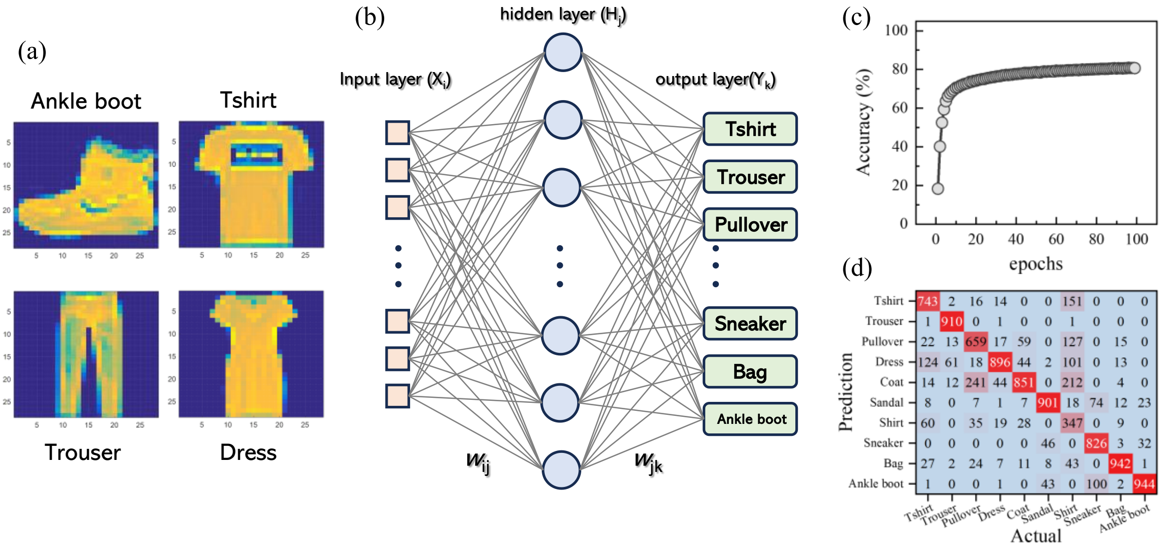

Figure S7. (a) Examples of input images of MNIST-fashion dataset composed as 28 by 28 pixels. (b) A schematic of neural networks (NNs) for MNIST-fashion recognition. (c) Recognition accuracy of NNs made using activation function from CSO:Tb/Al, as a function of epochs. (d) Confusion matrix of NNs made using activation function from CSO:Tb/Al after 100 epochs.

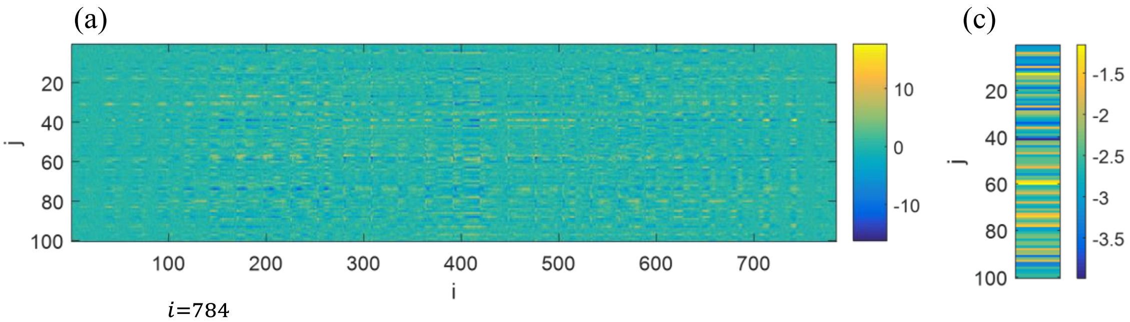

$$H_j = f(\sum_{i=1}^{i=784} (w_{ij} + b_j))$$

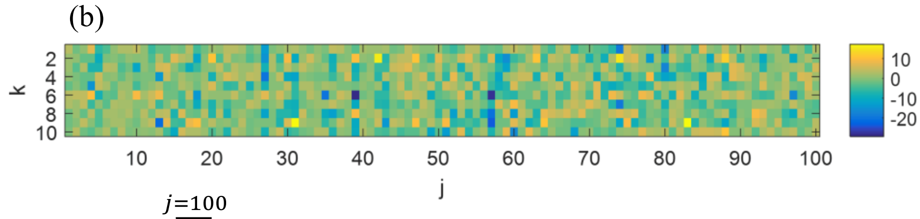

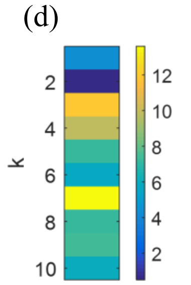

$$Y_k = f(\sum_{j=1}^{j=100} (w_{jk}+b_k))$$

Figure S8. The weight matrix of the connection (a) between input layer and hidden layer (w_{ij}), and (b) between hidden layer and output layer (w_{jk}). The bias of the connection (c) between input layer and hidden layer (b_j), and (d) between hidden layer and output layer (b_k) from NN for MNIST fashion recognition with 784×100×10 nodes, after 100 epochs. The expressions for H_j and Y_k are representing the signal propagation in processing of NN.

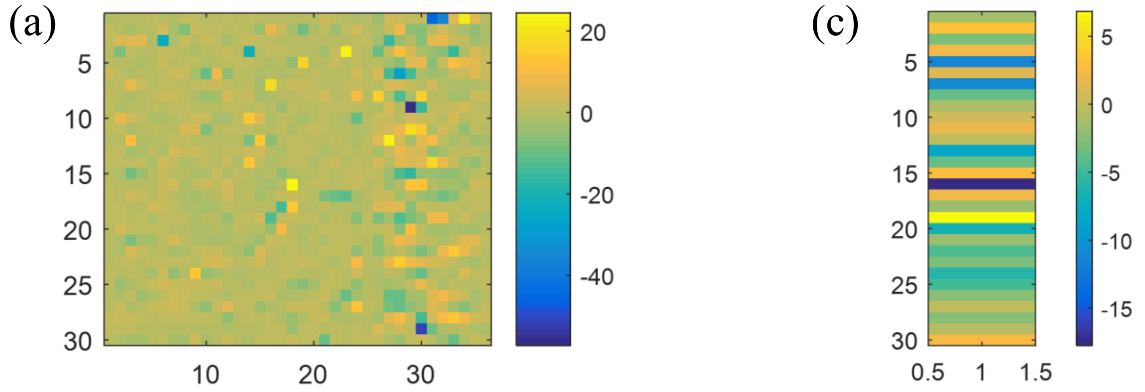

$$H_j = f(\sum_{i=1}^{i=36}(w_{ij} + b_j))$$

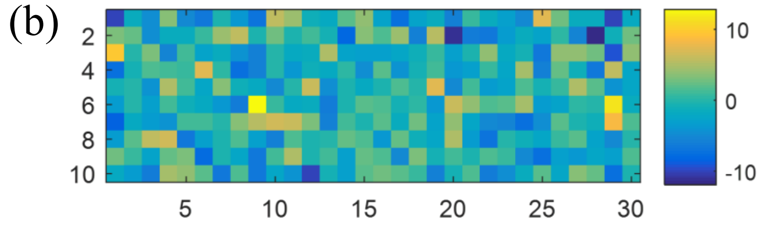

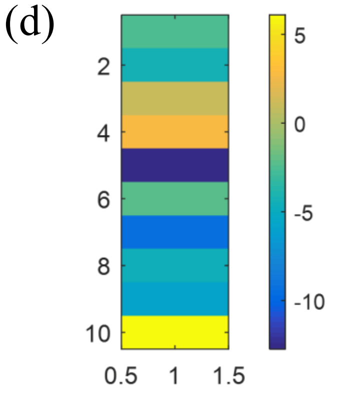

$$Y_k = f(\sum_{j=1}^{j=30}(w_{jk} + b_k))$$

Figure S9. The weight matrix of the connection (a) between input layer and hidden layer (w_{ij}), and (b) between hidden layer and output layer (w_{jk}). The bias of the connection (c) between input layer and hidden layer (b_j), and (d) between hidden layer and output layer (b_k) from NN with LPL driven RC for MNIST handwritten recognition with 36×30×10 nodes, after 100 epochs. The expressions for H_j and Y_k are representing the signal propagation in processing of NN.
